# Milk free of A1 β-casein supports superior gains in cognition and quality of life, relative to conventional milk, in older adults with mild cognitive impairment

**DOI:** 10.1016/j.jnha.2025.100579

**Published:** 2025-05-14

**Authors:** Keming Zhang, Jianqin Sun, Mingming Han, Yingfei Diao, Yan Xia, Congqing Yang, Stephen R. Robinson

**Affiliations:** a First Teaching Hospital of Tianjin University of Traditional Chinese Medicine, Tianjin, China; b Huadong Hospital, Fudan University, Shanghai, China; c RMIT University, Bundoora, Victoria, Australia; d Institute for Breathing and Sleep (IBAS), Austin Health, Heidelberg, Victoria, Australia

**Keywords:** A2 milk, A1 protein-free milk, Beta casein, Handgrip strength

## Abstract

**Objectives:**

To determine whether the regular consumption of milk free from A1 β-casein (A1PF milk) improves cognitive performance to a greater extent than conventional milk, and if so, whether such improvements are associated with an increase in the serum titres of reduced glutathione (GSH).

**Design:**

A multi-centre, double-blind, randomised controlled trial with two parallel arms, conducted from 7 March 2023 to 13 October 2023.

**Setting:**

Two hospitals in Tianjin, China.

**Participants:**

Volunteers (N = 96) diagnosed with mild cognitive impairment (MCI) and aged between 65 and 75 years.

**Intervention:**

A1PF skim milk powder or conventional skim milk powder, diluted into liquid form (200 mL) and consumed twice daily for 90 days.

**Measurements:**

The primary outcomes were cognitive performance (assessed with the Subtle Cognitive Impairment Test [SCIT]) and serum titres of GSH. Secondary outcomes included performance on two other cognitive tests, serum levels of 25-hydroxyvitamin D3, subjective quality of life (QoL), muscle strength (hand grip scale), faecal and blood inflammatory markers.

**Results:**

Data from 91 participants were analysed (A1PF milk group: n = 45; conventional milk group: n = 46). A1PF milk improved performance on all three cognitive tests to a greater extent than conventional milk; however, this improvement was not associated with an increase in serum GSH. When compared with conventional milk, A1PF milk resulted in higher increases in serum levels of 25-hydroxyvitamin D3, greater subjective improvements in QoL and improved left hand grip strength. There were no between-group differences in inflammatory markers, calcium absorption or bone density markers.

**Conclusion:**

Daily intake of A1PF milk for 90 days significantly improved cognition, QoL and muscle strength in a sample of older people with MCI. While these outcomes appear to be linked to increased serum titres of 25-hydroxyvitamin D3, further investigations are needed to confirm this association.

**Clinical trial registration:**

NCT05741047 (clinicaltrials.gov).

## 1 Introduction

In many parts of the world, especially in Europe and North America, dairy is a widely consumed dietary staple. Dairy consumption varies across China, although there has been a notable increase among older adults in recent years [1]. Cows’ milk can benefit older adults because of its high protein and nutrient composition, including calcium and vitamin D. Since the regular consumption of cows' milk protein by older adults increases muscle mass and possibly facilitates bone metabolism, it is considered beneficial for the management of sarcopaenia [2,3].

A large proportion (∼30%) of the protein in cows’ milk is β-casein, and 12 genetic variants of β-casein have been reported. These variants can be categorised as A1- or A2-type β-casein based on the presence of the amino acid substitution at position 67 [4]. The A2-type β-casein is the original form of β-casein and retains a proline residue at position 67, whereas the A1-type β-casein, which arose following the domestication of cattle, has a histidine residue [5]. This amino acid change results in a difference in the capacity and rate of these β-casein types to be broken down into β-casomorphin-7 (BCM-7): the A1-type of β-casein is easily cleaved to form BCM-7, but digestion of A2-type β-casein results in very little or no BCM-7 [6,7].

BCM-7 has opioid-like activity, and can stimulate (or suppress, depending on the BCM-7 concentration) T lymphocyte proliferation, reduce cysteine uptake, decrease the production of reduced glutathione (GSH), and increase oxidative stress [[8], [9], [10]]. These negative effects of BCM-7 can be avoided by consuming milk that is A1 β-casein-protein-free (A1PF milk), which is generally better tolerated by persons with an intolerance to milk [[11], [12], [13], [14]]. Randomised studies have shown that consumption of milk containing both A1- and A2-type β-caseins (conventional milk) is associated with significantly more abdominal pain and looser stool consistency than when consuming A1PF milk [15].

In children and adults who are intolerant of A1 β-casein, regular consumption of conventional milk is associated with poorer cognitive performance than when A1PF milk is regularly consumed [16,17]. This association appears to be driven by higher titres of inflammatory cytokines in the serum, which are thought to originate from bowel inflammation caused by exposure to BCM-7. Animal studies have demonstrated that cytokines originating from an inflammed bowel can stimulate leakiness of the blood-brain barrier thereby provoking neuroinflammation and influencing neuronal function [18].

Individuals who naturally tolerate cows’ milk do not experience significant levels of bowel inflammation after consuming conventional milk, and may derive cognitive and physical benefits from the regular consumption of milk [[19], [20], [21], [22]]. While the physiological basis of these cognitive benefits is unclear, a potential mechanism is suggested by the fact that milk consumption increases the concentration of GSH in the brain [23,24]. This antioxidant plays an essential role in protecting neurones from oxidative stress, including that caused by neuroinflammation [25].

Until now, the effects of milk consumption on cognition have been examined with conventional milk only. However, consumption of A1PF milk has been shown to increase plasma GSH concentrations to a significantly greater extent than conventional milk [16,26], which raises the interesting possibility that A1PF milk may have a more beneficial effect on cognition. To investigate this possibility, we compared the regular consumption of conventional and A1PF milk to determine whether they provide the same levels of benefit for cognition, muscle strength and quality of life (QoL). Community-dwelling Chinese citizens with mild cognitive impairment (MCI) and with an established tolerance for cows’ milk were selected because previous studies have shown that this demographic responds positively to milk supplementation [27,28]. The primary outcome measures in this double-blind randomised controlled trial were cognitive performance, as measured by the Subtle Cognitive Impairment Test (SCIT) [29], and titres of serum GSH. The secondary outcome measures included performance on other cognitive tests, serum titres of 25-hydroxyvitamin D3, QoL and hand grip strength, serum inflammatory markers, and serum markers of bone density.

## 2 Materials and methods

### 2.1 Study design

This was a multi-centre, double-blind, randomised controlled trial with two parallel arms and was conducted at two sites in China (the First Affiliated Hospital of Tianjin University of Traditional Chinese Medicine and Tianjin Medical University General Hospital) from 7 March 2023 to 13 October 2023.

**Fig. 1:**
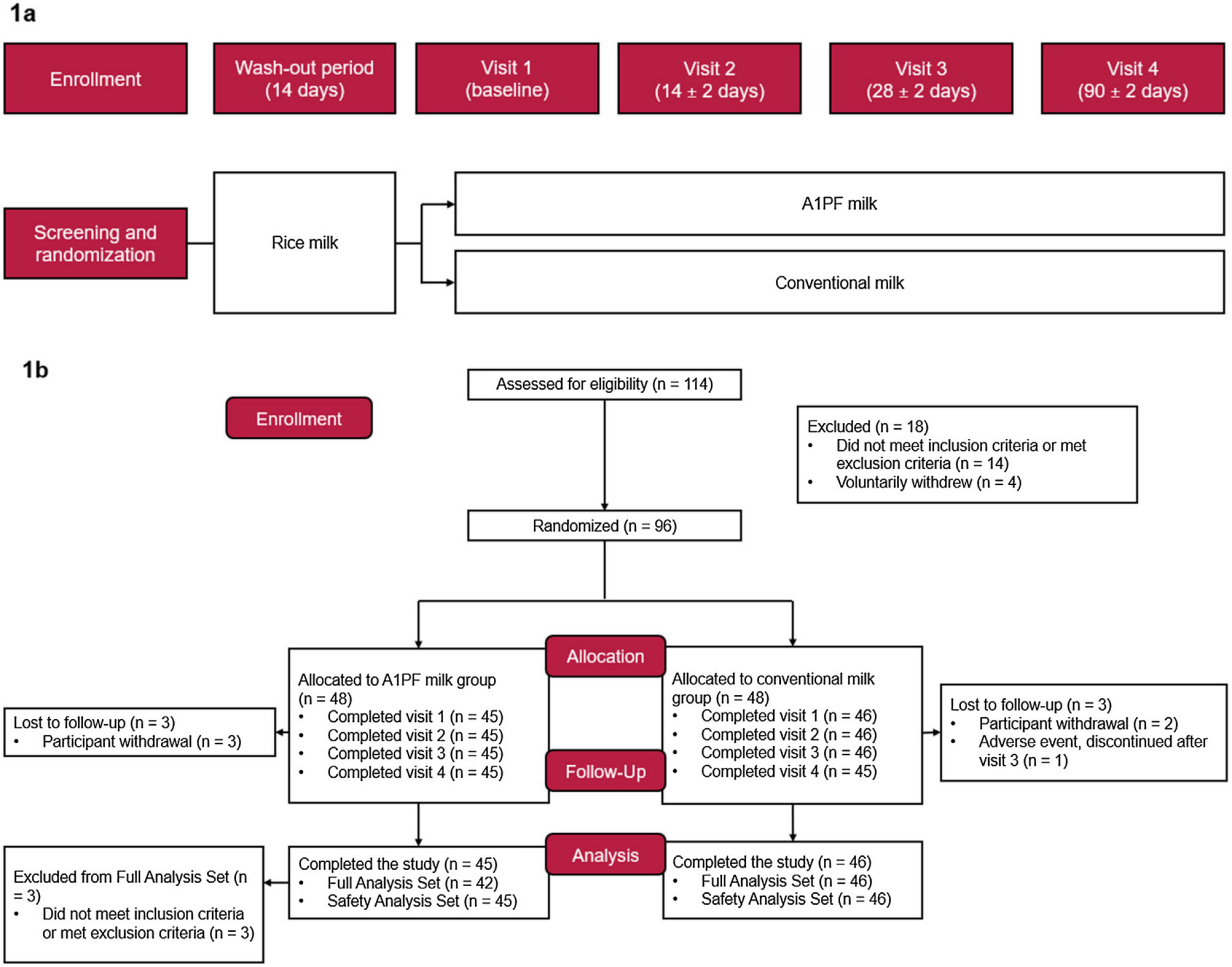
**1a.** Study design and **1b.** Participant disposition. Participant withdrawals (n = 3 in the A1PF milk group and n = 2 in the conventional milk group) were because of participants not consuming the study powdered milk.

Participants aged between 65 and 75 years were screened for eligibility and randomised to receive skim A1PF milk or skim conventional milk powder. The study lasted 104 days, consisting of a 14-day washout period prior to 90 days of milk between baseline (Visit 1) and Visit 4. All participants consumed rice milk during the washout period until Visit 1 (Fig. 1a). There were four visits in total: Visit 1 (baseline), 14 ± 2 days after enrolment; Visit 2: 14 ± 2 days from baseline; Visit 3: 28 ± 2 days from baseline; and Visit 4: 90 ± 2 days from baseline. A post-screening evaluation was conducted to ensure records were maintained, data were de-identified, Case Report Forms were completed, and adverse events (AEs) were recorded. Cognitive function, inflammation markers, dietary intake, QoL, handgrip strength, and the incidence of medically confirmed AEs were also recorded and compared.

The trial was registered at Clinicaltrials.gov under the identifier NCT05741047. The study adhered to the principles laid down in the latest version of the Declaration of Helsinki and Good Clinical Practice Guidelines, and local laws and regulations, including the International Conference on Harmonisation of Technical Requirements for Registration of Pharmaceuticals for Human Use. The informed consent form and associated documents were approved by the main site’s Research Ethics Committee and Human Genetic Resources Administration of China. All participants provided written informed consent to participate in the study at enrolment. Participants received the skim milk powders at no cost throughout the study, and reasonable travel expenses for visits beyond standard care were reimbursed. No additional benefits were provided.

### 2.2 Participants

The eligibility criteria were community-dwelling adults aged between 65 and 75 years (inclusive), with reported memory loss for >6 months, with cognitive ability scores lower than the standard cut-off value according to their age and education level on the Chinese version of the Mini-Mental State Examination (MMSE; MMSE score ≤17, ≤20, and ≤24 points if years of education are zero, ≤6, and >6, respectively), with an activities of daily living score ≤18, who did not meet the diagnostic criteria for major neurocognitive disorder (Diagnostic and Statistical Manual of Mental Disorders - Fifth Edition [30]) or Alzheimer’s Disease (National Stroke Institute for Neuropathic Speech Disorders and Association for Alzheimer Disease and Related Disorders), and who agreed not to participate in another interventional clinical research study during the present study. Consequently, participants enrolled in this study were deemed to meet the criteria for MCI [31], which is also known as mild neurocognitive disorder [30].

The exclusion criteria were known mental disorders, traumatic brain damage, or other physical disorders that can lead to cognitive impairment; neurological examination showing focal signs of central nervous system disorder, including hemiplegia, dysesthesia, aphasia; history of cerebrovascular diseases (including haemorrhagic and ischaemic types), internal brain trauma or fracture; asthmatic bronchitis, severe hypertension, angina, or severe infection; mental disorders, including depression and anxiety; endocrine system diseases, including hyperthyroidism, hypothyroidism, systemic lupus erythematosus, and rheumatoid arthritis; newly diagnosed, progressing, or advanced tumours; visual, reading, or hearing impairment or language communication difficulties that affected cognitive function testing; history of alcohol dependence or abuse of psychoactive substances (e.g., antipsychotics, benzodiazepines, cholinesterase inhibitors, or sedatives) or use of drugs that affect cognitive function; neurological diseases (e.g., Parkinson's disease or epilepsy); antibiotic treatment in the previous 2 weeks; immunosuppressive drugs in the 4 weeks preceding screening; milk or dairy allergies; diagnosed lactose intolerance; or other diseases that the investigators judged as unsuitable to participate in the study.

### 2.3 Randomisation and blinding

Participants were randomly assigned to receive either A1PF milk or conventional milk using block randomisation based on a pre-determined randomisation schedule. The A1PF skim milk powder and conventional skim milk powder packages were fully covered by tear-proof stickers to ensure blinding. Neither the trial team nor the participants were unblinded at any time during the course of the study.

### 2.4 Study milk

Participants received skim A1PF milk powder (skim a2 Milk™ powder [The A2 Milk Company, New Zealand]) or a conventional skim milk powder (skim milk powder [Yili Group, China]) that was available on the China market. Participants consumed the powder twice daily, dissolved in water based on the instructions on the labels: 200 mL during breakfast and another 200 mL before bed, for a total of 90 days.

### 2.5 Prior and concomitant therapy

During the 90-day trial period, no dairy products other than the assigned milk were consumed. Participants were discontinued from the trial if the investigators deemed it necessary, including if the participant consumed any other dairy during the trial period.

### 2.6 Study outcomes

The primary outcomes were the effect of A1PF milk and conventional milk on cognitive impairment as measured by the SCIT, and titres of serum GSH.

Secondary outcomes included additional cognitive function measures (the Montreal Cognitive Assessment [MoCA] and Auditory Verbal Learning Test [AVLT]). For QoL, we assessed Healthy Brain Ageing – Functional Assessment Questionnaire (HBA-FAQ) (Supplementary Text), muscle strength using the hand grip scale, faecal inflammatory markers (calprotectin [S100A8/A9]), myeloperoxidase, and short-chain fatty acids (SCFAs), blood inflammatory markers (C-reactive protein [CRP]), interleukin (IL)-1β, IL-4, IL-8, tumor necrosis factor (TNF)α, immunoglobulin (Ig)E, IgG1, IgG2A, and serum BCM-7), serum calcium and hydroxyvitamin D3, C-terminal telopeptide of type 1 collagen (CTX) and osteocalcin and urinary galactose.

Participants completed a food diary, recording intake of trial milk, health supplements, medications, and foods high in aluminium (churros, fritters, and puffed food), as well as digestive discomfort.

### 2.7 Safety

Throughout the trial, AEs were recorded as a measure of tolerability and safety.

### 2.8 Data collection

Stool samples were obtained from all participants at each visit (including baseline). Blood samples were collected at baseline, Visit 2, and Visit 4. Urine samples were collected at baseline and Visit 4.

SCIT tests were performed at baseline, Visit 2, and Visit 4 using computers at the participating hospitals. De-identified SCIT scores were analysed by a blinded investigator.

AVLT, MoCA, and QoL (HBA-FAQ) were assessed at baseline, Visit 2, and Visit 4 using paper questionnaires administered by investigators to the participants.

### 2.9 Statistical analysis

A target sample size of 96 participants was planned (48 participants randomised 1:1 in each arm) to detect a difference in change in SCIT response times at a 5% significance level with 80% power, accounting for 20% attrition. The achieved sample size was 96 (1:1 in each arm); 91 participants were included in the Safety Analysis Set (SAS), and 88 participants (42 randomised to the A1PF milk group and 46 to the conventional milk group) were included in the Full Analysis Set (FAS). Because the present paper is concerned with the longer-term effects of milk consumption, the data and analyses presented here are for Visit 1 (baseline) and Visit 4 only.

All data are presented as descriptive statistics (number [N], arithmetic mean, least squares mean, standard deviation [SD] or standard error, 95% confidence interval [CI], median, interquartile range, minimum, maximum) or frequency as appropriate. Normality testing was conducted using the Shapiro–Wilk test. Unadjusted pairwise comparisons of normally distributed continuous data were conducted using a two-tailed independent-samples t-test, and non-normally distributed continuous data were compared using a Wilcoxon rank-sum test. Categorical variables were compared using a chi-square test or Fisher’s exact test. Ordinal variables were compared using a Wilcoxon rank-sum test unless otherwise stated. Data from the same participant over timepoints (visit 1 vs visit 2, visit 1 vs visit 4, and visit 2 vs visit 4) were compared using a paired two-tailed Wilcoxon signed-rank test with continuity correction. The primary efficacy outcomes and the secondary efficacy outcomes were assessed using a mixed model of repeated measures (MMRM) with the PROC MIXED statement, which allows the inclusion of participants with missing data. Missing baseline and safety data were not imputed.

For all analyses, *p* ≤ 0.05 was considered significant. Faecal assays were conducted to an accuracy of three decimal places, except for calprotectin and myeloperoxidase, which were accurate to two decimal places; serum assays were accurate to two decimal places, except CRP, which was accurate to three decimal places. All assay values have been rounded to one decimal place for presentation purposes. An online calculator (https://www.wessa.net/rwasp_twosampletests_mean.wasp) was used to analyse the SCIT scores. SAS software version 9.4 or above (SAS Institute Inc., Cary, NC, USA) was used for all other data analysis.

## 3 Results

### 3.1 Participant disposition, baseline characteristics, and compliance

A total of 114 participants were assessed for eligibility; 18 were excluded (n = 14 did not meet the inclusion criteria or met the exclusion criteria, and n = 4 withdrew voluntarily). After screening and randomisation, 96 participants (48 in the A1PF milk group and 48 in the conventional milk group) were enrolled, with the first participant enrolled on 7 March 2023. Ninety participants completed all visits, and one participant discontinued after Visit 3 because of an AE unrelated to the study product. Ninety-one participants (A1PF milk group: n = 45; conventional milk group: n = 46) were included in the SAS, and 88 participants (A1PF milk group: n = 42, conventional milk group: n = 46) were included in the FAS. Five participants were excluded from the SAS and the FAS because they had not ingested any study milk. A further three participants were excluded from the FAS because of protocol violations (participants did not meet the inclusion criteria or met the exclusion criteria) (Fig. 1b).

Twenty-two women (52.4%) were included in the A1PF milk group and 27 (58.7%) in the conventional milk group. The mean (SD) age of participants was 69.0 (2.6) years in the A1PF milk group and 69.2 (2.6) years in the conventional milk group. None of the participants reported receiving hormones; most were non-smokers (A1PF milk group: 64.3%, conventional milk group: 82.6%) and non-drinkers (A1PF milk group: 66.7%, conventional milk group: 78.3%). No statistically significant differences in baseline characteristics were observed between A1PF and conventional milk group participants (Table 1). Only one participant (conventional milk group) did not meet the compliance criteria because of an unrelated AE, and there were no significant differences between the groups regarding compliance.

**Table 1:** Baseline characteristics, duration of skim milk use, compliance rates, and concomitant medications.

	A1PF milk (n = 42)	Conventional milk (n = 46)	*P* value
Sex			
Female	22 (52.4)	27 (58.7)	0.551^a^
Male	20 (47.6)	19 (41.3)	
Age, year			
Mean ± SD	69.0 ± 2.6	69.2 ± 2.6	0.822^b^
BMI, kg/m^2^			
Mean ± SD	25.9 ± 2.9	25.3 ± 3.5	0.359^c^
Medication history			
No	13 (31.0)	18 (39.1)	0.422^a^
Yes	29 (69.0)	28 (60.9)	
Sleep disorder			
No	20 (47.6)	20 (43.5)	0.643^b^
Mild	14 (33.3)	16 (34.8)	
Moderate	6 (14.3)	6 (13.0)	
Severe	2 (4.8)	4 (8.7)	
Serious	0 (0.0)	0 (0.0)	
Chronic pain			
No	21 (50.0)	15 (32.6)	0.153^b^
Mild	15 (35.7)	23 (50.0)	
Moderate	5 (11.9)	7 (15.2)	
Severe	1 (2.4)	1 (2.2)	
Serious	0 (0.0)	0 (0.0)	
Stress			
No	34 (81.0)	35 (76.1)	0.631^b^
Mild	4 (9.5)	7 (15.2)	
Moderate	4 (9.5)	3 (6.5)	
Severe	0 (0.0)	1 (2.2)	
Serious	0 (0.0)	0 (0.0)	
Depression			
No	33 (78.6)	37 (80.4)	0.793^b^
Mild	7 (16.7)	8 (17.4)	
Moderate	2 (4.8)	1 (2.2)	
Severe	0 (0.0)	0 (0.0)	
Serious	0 (0.0)	0 (0.0)	
Fatigue			
No	23 (54.8)	28 (60.9)	0.539^b^
Mild	9 (21.4)	9 (19.6)	
Moderate	5 (11.9)	5 (10.9)	
Severe	5 (11.9)	4 (8.7)	
Serious	0 (0.0)	0 (0.0)	
Diarrhea			
No	39 (92.9)	41 (89.1)	0.575^b^
Mild	1 (2.4)	3 (6.5)	
Moderate	2 (4.8)	2 (4.3)	
Severe	0 (0.0)	0 (0.0)	
Serious	0 (0.0)	0 (0.0)	
Hyperlipidemia			
No	24 (57.1)	28 (60.9)	0.722^a^
Yes	18 (42.9)	18 (39.1)	
Diabetes			
No	31 (73.8)	37 (80.4)	0.459^a^
Yes	11 (26.2)	9 (19.6)	
Smoking history			
Non-smoker	27 (64.3)	38 (82.6)	0.099^d^
Ex-smoker	7 (16.7)	2 (4.3)	
Smoker	8 (19.0)	6 (13.0)	
Alcohol consumption			
Non-drinker	28 (66.7)	36 (78.3)	0.401^d^
Ex-drinker	1 (2.4)	2 (4.3)	
Drinker	13 (31.0)	8 (17.4)	
Physical activity			
Never	20 (47.6)	13 (28.3)	0.090^b^
Sometimes	3 (7.1)	5 (10.9)	
Often	19 (45.2)	28 (60.9)	
Duration of powdered skim milk use, days			
Mean ± SD	91.5 ± 1.2	90.20 ± 9.3	0.902^b^
Study duration, days			
Mean ± SD	106.1 ± 2.1	106.02 ± 2.3	0.798^b^
Compliance rate, %			
Mean ± SD	100.9 ± 2.1	99.0 ± 10.6	0.990^b^
Compliance classification, n (%)			
<80%	0 (0.0)	1 (2.2)	1.000^b^
≥80% and ≤120%	42 (100.0)	45 (97.8)	
>120%	0 (0.0)	0 (0.0)	
Concomitant medication, n (%)			
No	22 (52.4)	18 (39.1)	0.212^a^
Yes	20 (47.6)	28 (60.9)	

Data shown as n (%) unless otherwise stated. Normally and non-normally distributed continuous data were compared using a two-tailed independent-samples t-test or a Wilcoxon rank-sum test. respectively. Categorical variables were compared using a chi-square test or Fisher's exact test. Ordinal variables were compared using a Wilcoxon rank-sum test unless otherwise stated.

The duration of powdered skim milk was the end date – the start date + 1. The study duration was the completion date/withdrawal date/discontinuation date – the date of informed consent + 1. The compliance rate was the actual number of times taken ÷ the theoretical number of times taken × 100.

BMI, body mass index; IQR, interquartile range; SD, standard deviation.

a Chi-square test.

b Wilcoxon rank-sum test.

c t-test.

d Fisher’s exact test.

### 3.2 Cognitive function (including changes in SCIT scores)

In the SCIT assessments (primary outcome), participants in both groups demonstrated numerically improved mean values for all SCIT parameters between baseline and Visit 4. However, only three parameters (response times in the head of the curve, mean errors, and errors in the head of the curve) showed a significant improvement between baseline and Visit 4 (Table 2). Interestingly, the A1PF group already showed a significant improvement at Visit 2 for both mean errors (*p* =  0.026), and errors in the head of the curve (*p* =  0.032), whereas the conventional milk group did not (*p* =  0.117 and *p* =  0.079, respectively). This suggests that the A1PF group responded more quickly to the milk supplementation than the conventional milk group.

**Table 2:** Summary data for SCIT performance.

	A1PF milk group	Conventional milk
**SCIT-RT, ms ± SD**		
Visit 1	651 ± 103	605 ± 130
Visit 4	608 ± 121	536 ± 110
*P* value (Visit 1 vs Visit 4)	0.179	0.273
**SCIT-RT_H_, ms ± SD**		
Visit 1	710 ± 115	669 ± 152
Visit 4	646 ± 123	582 ± 124
* P* value (Visit 1 vs Visit 4)	**0.018**	**0.029**
**SCIT-RT_T_, ms ± SD**		
Visit 1	593 ± 106	540 ± 112
Visit 4	569 ± 126	489 ± 101
* P* value (Visit 1 vs Visit 4)	0.455	0.542
**SCIT-E, % ± SD**		
Visit 1	34.3 ± 12.9	34.6 ± 15.9
Visit 4	26.5 ± 13.8	25.7 ± 12.3
* P* value (Visit 1 vs Visit 4)	**0.022**	**0.045**
**SCIT-E_H_, %**		
Visit 1	50.2 ± 13.9	47.7 ± 19.5
Visit 4	41.5 ± 17.6	38.1 ± 14.2
* P* value (Visit 1 vs Visit 4)	**0.033**	**0.045**
**SCIT-E_T_, %**		
Visit 1	18.5 ± 15.7	21.5 ± 17.2
Visit 4	11.6 ± 11.2	13.3 ± 13.3
*P* value (Visit 1 vs Visit 4)	0.116	0.117

Data are mean ± SD. Statistical significance assessed using a two-tailed Wilcoxon signed-rank test with continuity correction. Significant differences are shown in bold.

ms, millisecond; SCIT; Subtle Cognitive Impairment Test; SCIT-E, SCIT error rate; SCIT-E_H_, SCIT head error rate; SCIT-E_T_, SCIT tail error rate; SCIT-RT, SCIT response time; SCIT-RT_H_, SCIT head response time; SCIT-RT_T_, SCIT tail response time; SD, standard deviation.

**Fig. 2:**
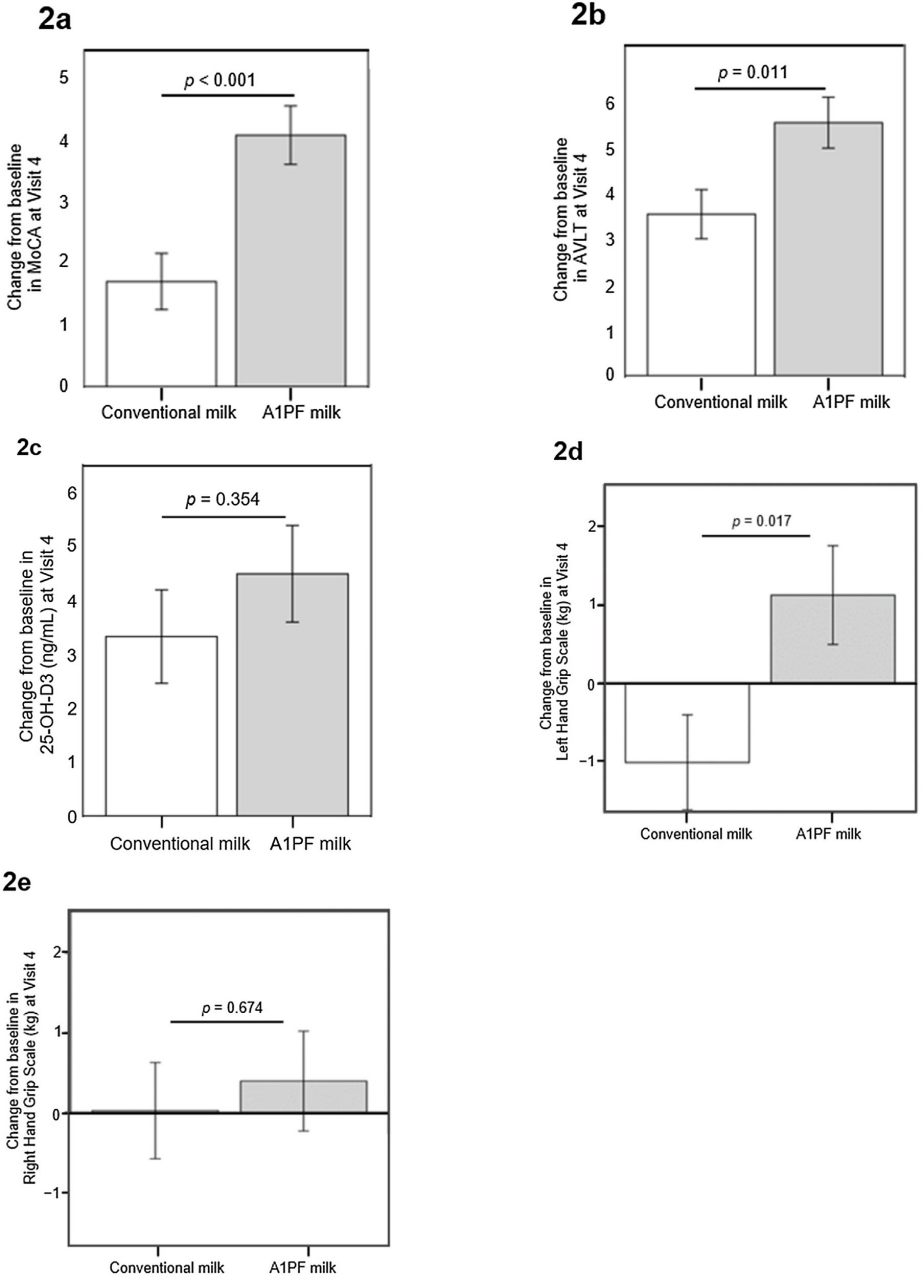
Change from baseline in cognitive function assessments with the **2a.** MoCa and **2b.** AVLT. **2c.** Change from baseline in 25-hydroxyvitamin D3. ​Change from baseline in hand grip strength in the **2d.** left hand and **2e.** right hand​. Data are shown as least squares mean of change from baseline (baseline values deducted), and error bars show standard error. AVLT, Auditory Verbal Learning Test; MoCA, Montreal Cognitive Assessment.

In the MMRM analysis, significant increases (improvements) in MoCA scores were seen in both groups at Visit 4 vs baseline (both groups *p* < 0.001). Significant increases in AVLT scores were also seen in both groups at Visit 4 vs baseline (all *p* < 0.001). Participants who consumed A1PF milk showed a significantly greater mean increase from baseline to Visit 4 in both MoCA scores and AVLT scores when compared with those who consumed conventional milk (MoCA score difference: 2.4 points [95% CI: 1.1, 3.7], *p* < 0.001 and AVLT score difference: 2.0 points [0.5, 3.6], *p* = 0.011, respectively) (Table 3, Fig. 2a and b).

**Table 3:** Change from baseline in cognition, hand grip strength and quality of life.

Variable	A1PF milk group	Conventional milk	Difference between means (95% CI) from Baseline at Visit 4	*P* value
**MoCA**				
Visit 1	19.9 ± 4.1	19.7 ± 3.8		
Visit 4	23.9 ± 3.2	21.4 ± 3.2	0.7 (1.1, 3.7)	**<0.001**
**AVLT**				
Visit 1	25.8 ± 4.3	25.8 ± 4.3		
Visit 4	31.4 ± 4.1	29.4 ± 4.1	0.8 (0.5, 3.6)	**0.011**
**Grip strength**				
Left-hand grip strength score, kg				
Visit 1	26.1 ± 8.9	23.9 ± 9.1		
Visit 4	26.9 ± 7.9	22.8 ± 8.1	2.1 (0.4, 3.9)	**0.017**
Right-hand grip strength score, kg				
Visit 1	27.2 ± 8.9	24.3 ± 9.0		
Visit 4	27.3 ± 8.2	24.3 ± 8.6	0.4 (−1.4, 2.1)	0.674
**Quality of Life**				
Self-care ability				
Visit 1	11.4 ± 0.8	11.2 ± 1.3		
Visit 4	13.1 ± 1.2	12.4 ± 0.8	0.7 (0.3, 1.1)	**0.002**
Financial handling capacity				
Visit 1	8.5 ± 1.3	8.4 ± 1.2		
Visit 4	9.9 ± 1.4	9.2 ± 0.7	0.6 (0.1, 1.1)	**0.010**
Interpersonal and social skills				
Visit 1	8.1 ± 1.3	8.4 ± 1.4		
Visit 4	10.2 ± 1.7	9.4 ± 1.0	0.8 (0.2, 1.4)	**0.012**
Housekeeping ability				
Visit 1	13.8 ± 2.3	13.7 ± 2.6		
Visit 4	16.6 ± 2.1	15.9 ± 1.6	0.7 (−0.1, 1.5)	0.099
Ability to use daily technology products				
Visit 1	8.6 ± 0.9	8.6 ± 0.9		
Visit 4	9.2 ± 0.8	9.1 ± 0.4	0.1 (−0.1, 0.4)	0.354
Sleep Quality				
Visit 1	13.9 ± 1.6	13.7 ± 1.9		
Visit 4	17.8 ± 2.6	16.7 ± 2.3	1.1 (0.0, 2.1)	**0.047**
Memory ability				
Visit 1	14.0 ± 2.6	14.2 ± 3.1		
Visit 4	20.1 ± 2.2	18.9 ± 2.0	1.2 (0.3, 2.1)	**0.011**
Higher level thinking skills				
Visit 1	13.7 ± 1.6	13.4 ± 2.2		
Visit 4	16.5 ± 2.2	15.5 ± 1.3	1.0 (0.2, 1.7)	**0.016**
Emotion management ability				
Visit 1	5.9 ± 1.0	5.8 ± 1.2		
Visit 4	6.9 ± 1.2	6.6 ± 0.9	0.3 (−0.2, 0.8)	0.208
Total				
Visit 1	97.9 ± 8.1	97.3 ± 11.7		
Visit 4	120.1 ± 10.3	113.7 ± 7.1	6.3 (2.6, 10.1)	**0.001**

Data are mean ± standard deviation, unless otherwise stated. Significant differences are shown in bold. *P* values were determined from the least squares mean change from baseline based on the analysis of covariance.

CI, confidence interval; AVLT, Auditory Verbal Learning Test; MoCA, Montreal Cognitive Assessment.

### 3.3 Changes in serum GSH

No significant changes from baseline were observed in titres of serum GSH (primary outcome) between the groups or within groups at any timepoints (Supplementary Table S1).

### 3.4 Faecal markers

In the MMRM analysis, there were no between-group differences in any change from baseline faecal measurements (Supplementary Table S1). Mean myeloperoxidase levels in the conventional milk group significantly decreased in participants at Visit 4 compared with baseline (difference: −1.0 ng/mL [−1.9, −0.1], *p* =  0.030).

### 3.5 Blood inflammatory markers

In the MMRM analysis, no significant differences were observed between the groups in the change from baseline at Visit 4 for any blood inflammatory markers (Supplementary Table S1).

### 3.6 Calcium absorption and vitamin D

In the MMRM analysis, no between-group differences were observed from baseline at Visit 4 for serum calcium measurements (Supplementary Table S1).

At Visit 4, participants in both groups experienced significant increases in 25-hydroxyvitamin D3 levels from baseline (both *p* < 0.001) (Supplementary Table S1), with an average increase of 4.5 ng/mL (95% CI: 2.7, 6.3) in the A1PF milk group and 3.3 ng/mL (95% CI: 1.6, 5.1) in the conventional milk group (Fig. 2c).

### 3.7 Bone density markers

The mean CTX and osteocalcin levels in the A1PF group at baseline were 0.4 ng/mL (SD: 0.2 ng/mL) and 15.4 ng/mL (4.8 ng/mL), respectively, and 0.4 ng/mL (SD: 0.2 ng/mL) and 16.3 ng/mL (6.0 ng/mL) in the conventional milk group. At visit 4, CTX levels were 0.3 ng/mL (0.1 ng/mL) in the A1PF group and 0.3 ng/mL (0.1 ng/mL) in the conventional milk group, and osteocalcin levels were 13.7 ng/mL (4.9 ng/mL) in the A1PF group and 14.5 ng/mL (5.4 ng/mL) in the conventional milk group. In the MMRM analysis, there were no between-group differences in the change from baseline at Visit 4 in CTX or osteocalcin levels. Participants in both groups had decreased mean CTX levels at Visit 4 vs baseline (A1PF milk group difference: −0.1 ng/mL [95% CI: −0.1, −0.1], conventional milk group difference: −0.1 ng/mL [−0.1, −0.1], both *p* < 0.001). Participants in both groups also had decreased mean osteocalcin levels at Visit 4 vs baseline (A1PF milk group difference: −1.9 ng/mL [−2.7, −1.0], conventional milk group difference: −1.8 ng/mL [−2.6, −0.9], both *p* < 0.001).

### 3.8 Hand grip strength

In the analysis of variance analysis, participants who consumed A1PF milk showed a significant improvement from baseline in left-hand grip strength score at Visit 4 compared with those who consumed conventional milk. Left-hand grip strength score increased from baseline in the A1PF milk group and decreased in the conventional milk group (mean change from baseline: 1.1 [95% CI: −0.1, 2.4] kg compared with −1.0 [−2.2, 0.2] kg, between-group difference in mean change from baseline: 2.1 [0.4, 3.9] kg; *p* = 0.017) (Table 3). There were no between-group differences in the change from baseline for right-hand grip strength scores (Fig. 2d and e).

### 3.9 Quality of life

The A1PF milk group had a significantly greater increase from baseline to Visit 4 in QoL total scores compared with the conventional milk group (mean change from baseline: 22.6 [95% CI: 19.9, 25.3] points compared with 16.3 [13.6, 18.9] points; between-group difference in mean change from baseline: 6.3 [2.6, 10.1] points, *p* = 0.001) (Table 3).

At Visit 4, participants in the A1PF milk group also had a greater increase from baseline in QoL subscale scores than the conventional milk group, including self-care ability (difference: 0.7 [95% CI: 0.3, 1.1] points, *p* = 0.002), financial handling capacity (0.6 [0.1, 1.1] points, *p* = 0.010), interpersonal and social skills (0.8 [0.2, 1.4] points, *p* =  0.012), sleep quality (1.1 [0.0, 2.1] points, *p* =  0.047), memory ability (1.2 [0.3, 2.1] points, *p* = 0.011), and higher level thinking skills scores (1.0 [0.2, 1.7] points, *p* = 0.016) (Table 3).

### 3.10 Urinary galactose

No statistically significant differences were observed over time or between groups in urinary galactose measurements.

### 3.11 Dietary intake

Significantly more participants in the A1PF milk group than in the conventional milk group reported a change in their intake of high-aluminum food at Visit 2 (9.5% vs 0.0%, *p* = 0.048), but no differences were observed at any of the other time points. Regarding health supplements and adherence to the protocol for milk intake, no differences were observed between the groups at any time point.

### 3.12 Tolerability

No AEs were recorded that were related to either milk type. At all time points, most participants did not report digestive discomfort (77.8%–97.8%). There were no between-group differences in any of the digestive discomfort measures at any of the time points.

## 4 Discussion

This was a double-blind, randomised, parallel, controlled trial of older community-dwelling adults with MCI. The primary aims were to determine whether regular consumption of A1PF milk improves cognitive performance to a greater extent than conventional milk, and if so, whether such improvements are associated with an increase in the serum titres of GSH. The data revealed that A1PF milk improved performance on all three cognitive tests compared with conventional milk, but unexpectedly, this improvement was not associated with an increase in serum GSH. However, as the ratio of GSH to oxidised glutathione (GSSG) was not analysed, it remains possible that there were changes in the relative proportions of reduced and oxidised glutathione. Furthermore, we found that A1PF milk resulted in higher serum levels of 25-hydroxyvitamin D3, greater subjective improvements in QoL and improved grip strength compared with conventional milk.

Both groups experienced significant increases in serum titres of 25-hydroxyvitamin D3 between baseline and Visit 4, with the A1PF group experiencing a mean increase of 4.5 ng/mL compared to 3.3 ng/mL in the conventional milk group. A1PF milk has also been reported to increase serum levels of vitamins B_3_ and B_12_ relative to conventional milk [32]. In that study, the difference was attributed to an inhibitory effect of BCM-7 on the absorption and bioavailability of vitamins B_3_ and B_12_ [32]. Therefore, it is possible that the lower serum levels of 25-hydroxyvitamin D observed in the conventional milk group in the present study are also due to the inhibitory effects of BCM-7 on absorption. Further research will be required to investigate this possibility.

One of the primary outcomes in the present study was cognitive performance. While diet, including the type of milk consumed, has been suggested to influence cognitive health [33,34], there are conflicting results as to the nature of the relationship between dairy consumption and cognition. Some papers indicate a lack of benefit or even an adverse effect on cognition [35,36], while others report a possible benefit with certain types of dairy [37]. It is possible that some of the negative outcomes reflect the consumption of milk or dairy by individuals with an intolerance to A1 β-casein or lactose, as these conditions provoke systemic inflammation that then impacts cognition [18,38]. In the present study, participants were excluded if they had overt symptoms of milk intolerance, and the success of this screening process was verified by the absence of significant increases in any serum or faecal inflammatory marker between baseline and Visit 4 in either group. This lack of an overt inflammatory response indicates that the participants were generally tolerant of milk and did not experience, to a significant degree, the negative effects that milk-induced bowel inflammation can have on cognition. However, we cannot preclude the possibility of subclinical effects on digestion and bowel function.

In this study, we observed significant improvements in cognitive performance, relative to baseline, following consumption of either conventional or A1PF milk by milk-tolerant adults. Moreover, we found that participants who consumed A1PF milk had a greater improvement in performance on three tests of cognition (SCIT, AVLT and MoCA). This outcome aligns with a double-blind cross-over study of Chinese school children that found that SCIT performance was significantly improved following 14 days of supplementation with A1PF milk [16]. The consistency of findings across different age groups suggests that A1PF milk may offer cognitive benefits across the life span.

At the outset of this study we had speculated that A1PF milk would confer a greater benefit on cognition because milk consumption increases brain concentrations of GSH [23,24], and A1PF milk boosts serum titres of GSH relative to conventional milk [16,26]. Furthermore, titres of GSH in serum and the brain decrease with age [39], so it seemed likely to us that the participants in this study would derive benefit from increases in GSH following consumption of A1PF milk. However, we observed no increases in the titres of serum GSH in either group, making it less likely that the cognitive benefits were attributable to increased brain levels of GSH. Nonetheless, as no assays were conducted to assess the ratio of reduced to oxidised glutathione [40], it remains possible that the ratio of GSH:GSSG was higher in the A1PF group, which may have contributed to the cognition improvements [41]. It is also possible that A1PF milk increased the availability of circulating L-cysteine, which is the rate-limiting step in the synthesis of GSH by the brain [42] and can upregulate the activity of glutathione peroxidase, which, in turn, increases the GSH:GSSG ratio [43]. In order to address these possibilities, future studies of this type may need to include serum assays for GSH:GSSG and L-cysteine.

A meta-analysis of data from 10,941 participants concluded that studies of dairy consumption in Western countries frequently report a lack of benefit for cognition, whereas studies conducted in South-East Asia commonly report a positive impact on cognition [21,27]. Wu and Sun suggested that there may be an upper limit to the benefit that can be received from dairy, and since dairy products represent a much smaller component of Asian diets than Western diets, this may present a greater opportunity for benefit [27]. One of the nutrients in milk is 25-hydroxyvitamin D3 [44]. Older people living in China are often severely deficient in 25-hydroxyvitamin D3 [45,46], so the regular intake of dairy products may help to address this deficiency. An inverse relationship is found between serum 25-hydroxyvitamin D3 levels and cognitive function, particularly in older adults [47,48]. Serum 25-hydroxyvitamin D3 can cross the blood–brain barrier where it contributes to brain function by assisting with the synthesis of neurotrophic factors, the modulation of calcium-binding proteins, and the inhibition of pro-inflammatory cytokines [49]. A recent meta-analysis of 24 clinical trials involving 7,557 participants reported that dietary supplementation with 25-hydroxyvitamin D3 results in small yet significant improvements in cognition, particularly in older adults and those who are deficient in vitamin D [50]. In the present study, the proportional increases in serum 25-hydroxyvitamin D3 are consistent with the comparative improvements in cognitive performance seen in the A1PF and conventional milk groups, suggesting that the cognitive improvements may be attributable to improved vitamin D status.

The present study found that grip strength in the left hand increased significantly between baseline and Visit 4 in the A1PF group, whereas the conventional milk group experienced no such improvement. In the A1PF group, the right hand also displayed an increase in grip strength, but this gain failed to reach statistical significance. Handgrip strength is a leading indicator of overall muscle strength [51]. Studies have shown that the regular consumption of milk improves muscle strength and reduces sarcopaenia in older adults, with this benefit often being attributed to milk proteins [52]. However, this interpretation has been challenged by a meta-analysis of 17 studies involving 1,544 older adults, which found that while milk diets consistently increased muscle mass, whey protein diets had no consistent effect [53]. Another nutrient in milk that has been linked to improved muscle mass and handgrip strength is vitamin D [51]. Serum levels of 25-hydroxyvitamin D3 are linearly related to muscle strength, and dietary supplementation with 25-hydroxyvitamin D3 stimulates the genesis of fast twitch type II muscle fibres [54]. These findings raise the possibility that the increased grip strength observed in the A1PF group is attributable to their increased titres of serum 25-hydroxyvitamin D3. This possibility, along with the relationships of other nutrients in milk, such as vitamin E [51], with grip strength, warrant further investigation.

The present study found a significant improvement in QoL in the A1PF group when compared to the conventional milk group, between baseline and Visit 4. Not only was this improvement evident in the overall QoL score, it was also evident in subscores relating to cognitive performance, sleep, self-care and interpersonal skills. While this outcome was unexpected, the subjective reports of improved memory, higher level thinking skills and financial handling capacity are consistent with the improved cognitive performance. Furthermore, the improved capacity for self-care may be attributable to increased muscle strength. It is notable that a cross-sectional study of 686 older adults in Northern China found that serum 25-hydroxyvitamin D3 levels were generally well below recommended levels, and that there was a highly significant linear correlation between vitamin D levels and QoL as measured by the 36-item Short Form Health Survey [46].

In the present study, mean serum 25-hydroxyvitamin D3 levels at baseline were 17.9 ng/mL (44.7 nmol) in both groups, which is indicative of severe vitamin D insufficiency [46]. By Visit 4, these levels had risen to 22.4 ng/mL (56.0 nmol) and 20.9 ng/mL (52.2 nmol) in the A1PF and conventional milk groups, respectively. Values between 50–75 nmol correspond to hypovitaminosis D [46]; therefore, despite the improvements in vitamin D status, participants in this study still had vitamin D levels below recommended levels by the end of the study. By extrapolating the rate of increase in serum 25-hydroxyvitamin D3 levels in the present study, it seems likely that participants would have continued to show improvements in cognition, QoL and hand grip strength if their milk intake had been continued for a further 3–6 months.

The present study has some limitations. First, serum titres of oxidised glutathione were not measured, nor were serum levels of cysteine, vitamin E or the B group vitamins. Since these nutrients have potential to improve cognition and/or muscle strength, it will be important to include assays for these markers in future studies so that their contributions can be understood. Second, as older persons with MCI have a heightened risk of progressing to Alzheimer's disease [55], a topic of considerable interest for future study is whether supplementation with A1PF milk can delay or slow the onset of Alzheimer's disease in people with MCI. For this reason, there is a need to replicate the study in a larger population and over longer periods of 6–12 months or more. Third, as the present study was conducted in a population that does not regularly consume large quantities of dairy, it would be valuable to explore whether similar benefits can be obtained in older persons who regularly consume conventional milk but not A1PF milk, or those with other dietary habits. Additionally, other dietary components may have influenced the observed results. Fourth, participants were contacted by the investigative team in both intervention arms, which may have positively influenced the self-reported QoL. Finally, it would be informative to include other measures of muscle mass and strength such as gait speed and dual-energy X-ray absorptiometry. Despite these limitations, the strengths of the study include its double-blind design, clear intervention, and overall good adherence by the participants to the study protocol.

In conclusion, the present results show that when compared to conventional milk, daily intake of A1PF milk for 90 days can significantly improve cognition, QoL and muscle strength in older people with MCI. While these outcomes may be linked to increased serum titres of 25-hydroxyvitamin D3, further investigations are needed to confirm this association.

## CRediT authorship contribution statement

**Keming Zhang:** Conceptualisation, Methodology, Formal Analysis, Investigation. **Jianqin Sun:** Conceptualisation, Methodology, Writing- Reviewing and Editing. **Mingming Han:** Investigation, Writing- Reviewing and Editing. **Yingfei Diao:** Investigation, Writing- Reviewing and Editing. **Yan Xia:** Investigation, Writing- Reviewing and Editing. **Congqing Yang:** Investigation, Writing- Reviewing and Editing. **Stephen R. Robinson:** Conceptualisation, Methodology, Formal Analysis, Investigation, Writing- Reviewing and Editing.

## Declaration of Generative AI and AI-assisted technologies in the writing process

Generative artificial intelligence (AI) and AI-assisted technologies were not used in the data acquisition, management, analysis, or writing of this manuscript.

## Funding

This research was funded by The a2 Milk Company.

## Data sharing

The data presented in this study are available from the corresponding author upon reasonable request.

## Declaration of competing interest

Stephen R. Robinson is a director of Neurotest Pty Ltd. This company markets the SCIT and also provides consultancy services for The a2 Milk Company. The other authors have no conflicts of interest to declare in relation to this study.

## Glossary

A1PF milk: A1 β-casein-protein-free milk
AE: Adverse event
AVLT: Auditory Verbal Learning Test
BCM-7: β-casomorphin 7
CI: Confidence interval
Conventional milk: Milk containing both A1- and A2-type β-caseins
CRP: C-reactive protein
CTX: C-terminal telopeptide of type 1 collagen
FAS: Full analysis set
GSH: Reduced glutathione
GSSG: Oxidised glutathione
HBA-FAQ: Healthy Brain Ageing – Functional Assessment Questionnaire
IG: Immunoglobulin
IL: Interleukin
MCI: Mild cognitive impairment
MMRM: Mixed model of repeated measures
MMSE: Mini-Mental State Examination
MoCA: Montreal Cognitive Assessment
QoL: Quality of life
SAS: Safety analysis set
SCFA: Short-chain fatty acid
SCIT: Subtle Cognitive Impairment Test
SD: Standard deviation
TNF: Tumor necrosis factor
