# Supplementary material for: Milk free of A1 β-casein supports superior gains in cognition and quality of life, relative to conventional milk, in older adults with mild cognitive impairment
Source: J Nutr Health Aging. 2025 May 14;29(7):100579. doi: 10.1016/j.jnha.2025.100579 (PMC12172951; doi:10.1016/j.jnha.2025.100579)
Supplement: Supplementary file 1 [file mmc1.docx]

**Supplementary materials**

**Supplementary Table 1.** **Change from baseline in faecal and blood inflammatory markers based on MMRM**

|  | Group | Visit time | Mean ± standard deviation | *P* value |
| --- | --- | --- | --- | --- |
| Serum GSH, µmol/L |  |  |  |  |
|  | A1PF milk | Visit 1 | 4.5 ± 0.6 |  |
|  | A1PF milk | Visit 4 | 4.5 ± 0.5 | 0.853 |
|  | Conventional milk | Visit 1 | 4.6 ± 0.5 |  |
|  | Conventional milk | Visit 4 | 4.6 ± 0.5 | 0.543 |
| Faecal  Calprotectin, ng/mL |  |  |  |  |
|  | A1PF milk | Visit 1 | 77.5 ± 12.1 |  |
|  | A1PF milk | Visit 4 | 77.5 ± 12.4 | 0.746 |
|  | Conventional milk | Visit 1 | 75.6 ± 16.1 |  |
|  | Conventional milk | Visit 4 | 75.3 ± 13.1 | 0.749 |
| Myeloperoxidase, ng/mL |  |  |  |  |
|  | A1PF milk | Visit 1 | 20.0 ± 4.1 |  |
|  | A1PF milk | Visit 4 | 19.5 ± 3.7 | 0.615 |
|  | Conventional milk | Visit 1 | 19.1 ± 3.9 |  |
|  | Conventional milk | Visit 4 | 18.3 ± 3.5 | **0.030** |
| Acetic acid, µmol/g |  |  |  |  |
|  | A1PF milk | Visit 1 | 2575.7 ± 791.7 |  |
|  | A1PF milk | Visit 4 | 2602.0 ± 936.9 | 0.909 |
|  | Conventional milk | Visit 1 | 2602.1 ± 735.4 |  |
|  | Conventional milk | Visit 4 | 2858.6 ± 1088.2 | 0.068 |
| Propionic acid, µmol/g |  |  |  |  |
|  | A1PF milk | Visit 1 | 978.9 ± 429.6 |  |
|  | A1PF milk | Visit 4 | 1039.2 ± 608.8 | 0.687 |
|  | Conventional milk | Visit 1 | 1060.7 ± 583.7 |  |
|  | Conventional milk | Visit 4 | 1066.6 ± 529.9 | 0.735 |
| Isobutyric acid, µmol/g |  |  |  |  |
|  | A1PF milk | Visit 1 | 221.1 ± 143.8 |  |
|  | A1PF milk | Visit 4 | 208.9 ± 120.9 | 0.694 |
|  | Conventional milk | Visit 1 | 160.8 ± 84.1 |  |
|  | Conventional milk | Visit 4 | 174.6 ± 97.2 | 0.865 |
| Butyric acid, µmol/g |  |  |  |  |
|  | A1PF milk | Visit 1 | 1236.8 ± 639.2 |  |
|  | A1PF milk | Visit 4 | 1159.8 ± 669.6 | 0.431 |
|  | Conventional milk | Visit 1 | 1223.3 ± 654.4 |  |
|  | Conventional milk | Visit 4 | 1134.0 ± 612.3 | 0.289 |
| Isovaleric acid, µmol/g |  |  |  |  |
|  | A1PF milk | Visit 1 | 153.4 ± 128.4 |  |
|  | A1PF milk | Visit 4 | 139.8 ± 98.4 | 0.734 |
|  | Conventional milk | Visit 1 | 98.3 ± 64.5 |  |
|  | Conventional milk | Visit 4 | 107.3 ± 78.0 | 0.551 |
| Valeric acid, µmol/g |  |  |  |  |
|  | A1PF milk | Visit 1 | 232.1 ± 187.0 |  |
|  | A1PF milk | Visit 4 | 211.5 ± 185.3 | 0.721 |
|  | Conventional milk | Visit 1 | 181.1 ± 155.6 |  |
|  | Conventional milk | Visit 4 | 165.3 ± 179.0 | 0.237 |
| Hexanoic acid, µmol/g |  |  |  |  |
|  | A1PF milk | Visit 1 | 47.4 ± 58.6 | 0.790 |
|  | A1PF milk | Visit 4 | 34.9 ± 38.7 | 0.072 |
|  | Conventional milk | Visit 1 | 44.9 ± 81.2 | 0.765 |
|  | Conventional milk | Visit 4 | 34.1 ± 55.7 | 0.118 |
| Blood  CRP, mg/L |  |  |  |  |
|  | A1PF milk | Visit 1 | 1.1 ± 0.3 |  |
|  | A1PF milk | Visit 4 | 1.0 ± 0.3 | 0.461 |
|  | Conventional milk | Visit 1 | 1.0 ± 0.3 |  |
|  | Conventional milk | Visit 4 | 1.0 ± 0.3 | 0.956 |
| IL-1β, pg/mL |  |  |  |  |
|  | A1PF milk | Visit 1 | 10.5 ± 12.8 |  |
|  | A1PF milk | Visit 4 | 10.8 ± 13.3 | 0.330 |
|  | Conventional milk | Visit 1 | 8.4 ± 8.5 |  |
|  | Conventional milk | Visit 4 | 9.3 ± 8.3 | 0.424 |
| IL-4, pg/mL |  |  |  |  |
|  | A1PF milk | Visit 1 | 5.8 ± 3.6 |  |
|  | A1PF milk | Visit 4 | 5.8 ± 2.4 | 0.986 |
|  | Conventional milk | Visit 1 | 5.5 ± 2.0 |  |
|  | Conventional milk | Visit 4 | 6.0 ± 2.1 | 0.114 |
| IL-8, pg/mL |  |  |  |  |
|  | A1PF milk | Visit 1 | 416.1 ± 245.2 |  |
|  | A1PF milk | Visit 4 | 426.5 ± 309.4 | 0.715 |
|  | Conventional milk | Visit 1 | 350.9 ± 197.6 |  |
|  | Conventional milk | Visit 4 | 426.5 ± 309.4 | 0.355 |
| TNF-α, pg/mL |  |  |  |  |
|  | A1PF milk | Visit 1 | 12.1 ± 13.9 |  |
|  | A1PF milk | Visit 4 | 12.1 ± 14.3 | 0.419 |
|  | Conventional milk | Visit 1 | 9.5 ± 8.8 |  |
|  | Conventional milk | Visit 4 | 10.6 ± 8.7 | 0.365 |
| IgE, ng/mL |  |  |  |  |
|  | A1PF milk | Visit 1 | 217.9 ± 62.6 |  |
|  | A1PF milk | Visit 4 | 204.6 ± 65.4 | 0.288 |
|  | Conventional milk | Visit 1 | 213.9 ± 64.3 |  |
|  | Conventional milk | Visit 4 | 210.1 ± 75.6 | 0.577 |
| IgG1, mg/mL |  |  |  |  |
|  | A1PF milk | Visit 1 | 1.9 ± 0.8 |  |
|  | A1PF milk | Visit 4 | 1.8 ± 0.7 | 0.347 |
|  | Conventional milk | Visit 1 | 2.6 ± 2.5 |  |
|  | Conventional milk | Visit 4 | 2.5 ± 3.0 | 0.739 |
| IgG2A, mg/mL |  |  |  |  |
|  | A1PF milk | Visit 1 | 594.2 ± 162.5 |  |
|  | A1PF milk | Visit 4 | 592.5 ± 164.0 | 0.848 |
|  | Conventional milk | Visit 1 | 677.2 ± 249.2 |  |
|  | Conventional milk | Visit 4 | 689.7 ± 324.2 | 0.767 |
| Calcium, mmol/L |  |  |  |  |
|  | A1PF milk | Visit 1 | 2.4 ± 0.1 |  |
|  | A1PF milk | Visit 4 | 2.4 ± 0.1 | 0.170 |
|  | Conventional milk | Visit 1 | 2.4 ± 0.1 |  |
|  | Conventional milk | Visit 4 | 2.4 ± 0.1 | 0.109 |
| 25-hydroxyvitamin D3, ng/mL |  |  |  |  |
|  | A1PF milk | Visit 1 | 17.9 ± 7.9 |  |
|  | A1PF milk | Visit 4 | 22.4 ± 7.6 | **<0.001** |
|  | Conventional milk | Visit 1 | 17.9 ± 7.5 |  |
|  | Conventional milk | Visit 4 | 20.9 ± 8.0 | **<0.001** |

Statistical significance shown in bold text. *P* values were determined from the least squares mean change from baseline based on the MMRM.

CRP, C-reactive protein; Ig, immunoglobulin; IL, interleukin; MMRM, mixed-effect models for repeated measures; TNF, tumor necrosis factor.

**Supplementary Text 1.** Chinese version of HBA-FAQ HBA Functional Assessment Questionnaire: self-report form.*

*(modified from Naismith & Mowszowski, 2014; Lin et al., 2022)

*We are interested to know whether you have noticed any changes in your daily functioning. Please complete this questionnaire according to* ***changes*** *you may have noticed within yourself over the last 3 months. Please answer every question to the best of your ability, circling* ***one*** *response for each item*.

1: Moderate change for the worse; 2: Mild change for the worse; 3: No change; 4: Mild change for the better; 5: Moderate change for the better.

| **Area of functioning** | | | | | |
| --- | --- | --- | --- | --- | --- |
| *Self-care* | | | | | |
| Getting ready in the morning or getting ready to go out (e.g., taking longer, disorganised, indecisive) | 1 | 2 | 3 | 4 | 5 |
| General self-grooming (e.g., poorer hygiene, less frequent showering, change in appearance) | 1 | 2 | 3 | 4 | 5 |
| Seeking medical and/or dental advice where warranted | 1 | 2 | 3 | 4 | 5 |
| Managing medications (e.g., taking medications as prescribed) | 1 | 2 | 3 | 4 | 5 |
| *Financial affairs* | | | | | |
| Handling money (e.g., difficulty dealing with cash or change at the shops) | 1 | 2 | 3 | 4 | 5 |
| Managing complex financial affairs (e.g., bank accounts) | 1 | 2 | 3 | 4 | 5 |
| Managing household finances (e.g., paying bills) | 1 | 2 | 3 | 4 | 5 |
| *Social functioning* | | | | | |
| Initiating and contributing to social activities with friends and/or family (e.g., birthdays/ outings) | 1 | 2 | 3 | 4 | 5 |
| Withdrawal (e.g., reduced motivation to engage in activities previously enjoyed) | 1 | 2 | 3 | 4 | 5 |
| Ease of using public transportation (e.g., getting confused or anxious when taking a trip on the bus or subway) | 1 | 2 | 3 | 4 | 5 |
| *Household tasks* | | | | | |
| Everyday household tasks such as vacuuming, cleaning, dusting, basic home maintenance | 1 | 2 | 3 | 4 | 5 |
| Keeping the house in order (e.g., cluttered/disorganised/dirty household, less ‘house- proud’) | 1 | 2 | 3 | 4 | 5 |
| Preparing meals (e.g., less initiative to plan/prepare meals, more use of microwave or takeaway) | 1 | 2 | 3 | 4 | 5 |
| Cooking (e.g., less effective (e.g., timing, taste, nutrition) at cooking a meal) | 1 | 2 | 3 | 4 | 5 |
| Grocery shopping (e.g., less effective at planning and carrying out grocery shopping) | 1 | 2 | 3 | 4 | 5 |
| *Everyday technology* | | | | | |
| Using the telephone (e.g., getting confused, making mistakes, avoiding making calls) | 1 | 2 | 3 | 4 | 5 |
| Using the computer or mobile phone (e.g., getting confused, making mistakes) | 1 | 2 | 3 | 4 | 5 |
| Using the remote control, television and video (e.g., getting confused, making mistakes) | 1 | 2 | 3 | 4 | 5 |
| *Sleep* | | | | | |
| Difficulty falling asleep (e.g., consistently taking more than 30 mins) | 1 | 2 | 3 | 4 | 5 |
| Waking during the night other than to go to the toilet (periods of wakefulness) | 1 | 2 | 3 | 4 | 5 |
| Change of napping habit (e.g. an increase in the number of daily naps or taking longer naps) | 1 | 2 | 3 | 4 | 5 |
| Changes in sleep timing (e.g., earlier or later bedtime, erratic sleep and wake times) | 1 | 2 | 3 | 4 | 5 |
| Acting out dreams (e.g., kicking, punching, hitting, yelling during sleep) | 1 | 2 | 3 | 4 | 5 |
| *Memory* | | | | | |
| Day-to-day memory problems (e.g., forgetting recent conversations, events, news items) | 1 | 2 | 3 | 4 | 5 |
| Being forgetful (e.g., forgetting where you have put your keys, losing or misplacing things) | 1 | 2 | 3 | 4 | 5 |
| Being repetitive in conversation | 1 | 2 | 3 | 4 | 5 |
| Word-finding problems (e.g., finding words in conversation, names of objects) | 1 | 2 | 3 | 4 | 5 |
| Confusion in unusual situations or environments | 1 | 2 | 3 | 4 | 5 |
| Forgetfulness during tasks (e.g., forgetting tasks mid-way, leaving the kettle or stove on) | 1 | 2 | 3 | 4 | 5 |
| *Higher-level thinking skills* | | | | | |
| Poorer judgment than usual (e.g., making bad or unusual decisions) | 1 | 2 | 3 | 4 | 5 |
| Difficulty making decisions (e.g., impulsive or irrational decisions, unable to make a decision) | 1 | 2 | 3 | 4 | 5 |
| Concrete thinking (seeing things as black or white, less appreciation of language subtleties) | 1 | 2 | 3 | 4 | 5 |
| Inflexible thinking (fixed on a topic, rigid ideas or opinions) | 1 | 2 | 3 | 4 | 5 |
| Becoming easily overwhelmed or slower thinking (e.g., when completing tasks) | 1 | 2 | 3 | 4 | 5 |
| *Emotion regulation* | | | | | |
| Irritability or temper outbursts | 1 | 2 | 3 | 4 | 5 |
| Mood changes (e.g., sadness, depression or lack of pleasure from previously enjoyable activities) | 1 | 2 | 3 | 4 | 5 |
